# Multiple components of PKA and TGF-β pathways are mutated in pseudomyxoma peritonei

**DOI:** 10.1371/journal.pone.0174898

**Published:** 2017-04-20

**Authors:** Lilli Saarinen, Pirjo Nummela, Alexandra Thiel, Rainer Lehtonen, Petrus Järvinen, Heikki Järvinen, Lauri A. Aaltonen, Anna Lepistö, Sampsa Hautaniemi, Ari Ristimäki

**Affiliations:** 1 Genome-Scale Biology Research Program, Research Programs Unit, University of Helsinki, Helsinki, Finland; 2 Department of Surgery, Helsinki University Hospital, Helsinki, Finland; 3 Department of Urology, Helsinki University Hospital, Helsinki, Finland; 4 Department of Medical Genetics, University of Helsinki, Helsinki, Finland; 5 Department of Pathology, HUSLAB, Helsinki University Hospital and University of Helsinki, Helsinki, Finland; Universita degli Studi di Torino, ITALY

## Abstract

Pseudomyxoma peritonei (PMP) is a subtype of mucinous adenocarcinoma mainly restricted to the peritoneal cavity and most commonly originating from the appendix. The genetic background of PMP is poorly understood and no targeted treatments are currently available for this fatal disease. While RAS signaling pathway is affected in most if not all PMP cases and over half of them also have a mutation in the *GNAS* gene, other genetic alterations and affected pathways are, to a large degree, poorly known. In this study, we sequenced whole coding genome of nine PMP tumors and paired normal tissues in order to identify additional, commonly mutated genes and signaling pathways affected in PMP. These exome sequencing results were validated with an ultra-deep amplicon sequencing method, leading to 14 validated variants. The validated results contain seven genes that contribute to the protein kinase A (PKA) pathway. PKA pathway, which also contains *GNAS*, is a major player of overproduction of mucin, which is the characteristic feature of PMP. In addition to PKA pathway, we identified mutations in six genes that belong to the transforming growth factor beta (TGF-β) pathway, which is a key regulator of cell proliferation. Since either *GNAS* mutation or an alternative mutation in the PKA pathway was identified in 8/9 patients, inhibition of the PKA pathway might reduce mucin production in most of the PMP patients and potentially suppress disease progression.

## Introduction

Pseudomyxoma peritonei (PMP) is a rare subtype of intestinal-derived mucinous adenocarcinoma, with prevalence of approximately 1–2 persons per million per year [1]. PMP originates most frequently from the appendix, and through its rupture the tumor cells spread into the peritoneal cavity. A characteristic feature of PMP is the ability of tumor cells to produce large amounts of extracellular mucus, which leads to bowel obstruction, breathing difficulties, malnutrition, and eventually death of the patient. According to the World Health Organization (WHO) classification PMP is categorized into low-grade (LG) and high-grade (HG) disease [2]. LG PMP is a relatively slowly progressing disease with 5-year survival of 63%, while the HG type of the disease has the ability to invade and metastasize, leading to 5-year survival of only 23% [3]. Current standard treatment of PMP is aggressive cytoreductive surgery combined with hyperthermic intraperitoneal chemotherapy [4]. This combinatory treatment, however, causes morbidity and mortality, and is only amenable for 60–70% of the patients [5]. Furthermore, as a significant proportion of PMPs usually relapse, additional targeted treatments are needed.

The genetic background of PMP is poorly understood due to its low incidence and challenges related to collection of representative tissue material of this remarkably mucinous tumor type. The published next-generation sequencing (NGS) studies [6–11] have mostly used targeted sequencing of mutational hotspot areas of known cancer related genes. These studies have discovered the characteristic *KRAS* and *GNAS* alterations in PMP, together with a lower frequency of e.g. *SMAD4*, *SMAD2*, *SMAD3*, *TP53*, *PIK3CA*, and *AKT1* mutations. Exome sequencing enables identification of variants in the whole coding region of the human genome and allows to expand the analysis of the mutations to pathway level.

In our previous study covering 212 somatic mutation hotspots in 48 cancer related genes in 19 patients [9], we found that all sequenced PMP tumors had an activating *KRAS* mutation and 12/19 (63%) of the patients harbored a mutation in the *GNAS* gene. Here, we have sequenced the whole coding genome of nine of these PMP tumors and paired normal tissues in order to identify common mutated genes and pathways outside the scope of the targeted hotspot panels. Mutations in the protein kinase A (PKA) pathway were under special focus, since PKA pathway activation is involved in the overproduction of mucin [7], and 4/9 tumors in our sample set lacked the typical *GNAS* mutation, which led to hypothesis that mutations in other genes in PKA pathway may lead to similar phenotype. Exome sequencing results were validated with an independent measurement method that is based on ultra-deep amplicon sequencing.

## Materials and methods

### Patients

Nine PMP tumor samples (6 LG and 3 HG) with histologically confirmed appendiceal origin were analyzed with exome sequencing. The tumor cell content of the samples was between 10% and 60% as estimated from hematoxylin and eosin stained (HE) slides. Nine matching control samples (from skin or gallbladder) were included into the analysis. Tissue samples were processed at Division of Pathology and Genetics, HUSLAB, at the Helsinki University Central Hospital between 2006 and 2013. Grading of the tumor samples was done according to the WHO 2010 classification [2]. This study was approved by the Ethics Committee of the Helsinki University Central Hospital (code 408/13/03/03/2009) and the patients gave written informed consent.

### DNA extraction

From the formalin-fixed paraffin-embedded (FFPE) tissue specimens, 10 μm flakes were cut with a Leica SM2000R microtome (Leica Microsystems GmbH, Wetzlar, Germany). Macrodissection was used when applicable to increase the yield of tumor DNA. Two to fifteen tissue flakes were deparaffinized and genomic DNA was extracted using QIAamp DNA Mini Kit (Qiagen, Venlo, the Netherlands) as previously reported [9]. DNA quality was inspected with NanoDrop spectrophotometer (Thermo Fischer Scientific, Waltham, MA) and agarose gel electrophoresis (with Midori Green Advanced DNA Stain; Nippon Genetics EUROPE GmbH, Dueren, Germany), and DNA concentration was determined using Qubit dsDNA HS Assay Kit and Qubit 2.0 Fluorometer (Molecular Probes/Life Technologies, Paisley, UK).

### Exome sequencing

Target enrichment for exome sequencing was done with the Agilent SureSelect 51M Capture Kit (Santa Clara, CA). Paired-end sequencing with 90 base pair read length was performed with Illumina HiSeq 2000 (San Diego, CA). Alignment of the reads was done by BWA [12] with parameters bwa aln -o 1 -e 50 -m 100000 -t 4 -i 15 -q 10 -l, and the human genome build 37 (hg19) was used as the reference. Duplicate reads were removed with Picard Tools (http://picard.sourceforge.net).

### Data analyses

**Fig 1:**
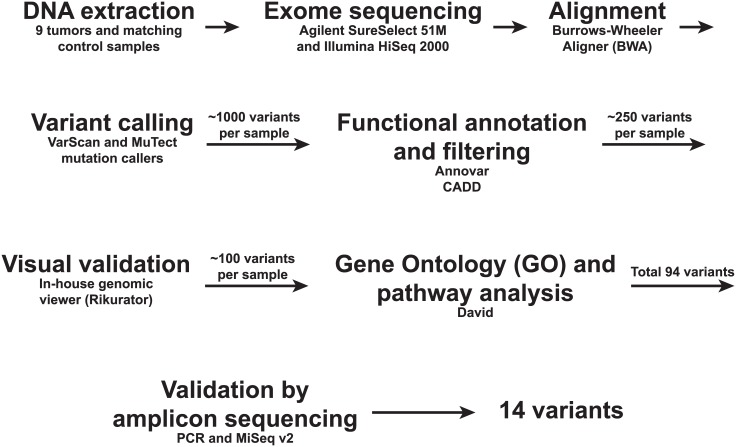
Workflow used for exome sequencing of nine pseudomyxoma peritonei specimens and their matching control samples.

Sequence data were analyzed with the Anduril workflow framework [13] (Fig 1). Variants were called by VarScan somatic (v.2.3.6) [14] and MuTect (v1.1.4) [15] in tumor-normal pairs. The pileup files for VarScan somatic were created using SAMTools mpileup (v0.1.19) [16] with default parameters except for -q 1 -Q 10. We estimated tumor cell percentage for each sample both visually from the HE stained slides and from the variant allele frequencies (VAF) of *KRAS* and *GNAS* variants obtained from our previous targeted deep sequencing of the same samples [9] (Table 1). The average read coverage of the targeted sequencing was 3,580X, making the VAFs and, thus, tumor cell percentages, more reliable than using VAFs from exome sequencing (average coverage 120X). Tumor purity estimated from the targeted sequencing, ranging from 6% to 28%, was then used as the purity parameter for each tumor sample in the VarScan variant caller algorithm. Other default parameters for VarScan were used except—min-avg-qual 10. VarScan derived somatic single nucleotide variation (SNV) and short insertion and deletion (indel) calls were filtered with Fisher test *p*-value < 0.05 to obtain statistically significant calls. VarScan results were further filtered to contain cancer related genes from the Cancer Gene Census [17] in addition with kinome genes [18] to prioritize functionally relevant calls. SNV calls from MuTect were not filtered, since the method itself contains stringent filtering, and allowed thus calls from the whole exonic regions. In order not to miss true positives we preferred sensitivity over specificity and combined the calls from VarScan and MuTect. The increase in false positives in the discovery set is overcome by the use of independent validation method. Synonymous changes were disregarded. Resulting variants were annotated with Annovar [19] and CADD (v1.2) functional scores [20]. Scaled CADD score > 10 was used to gain the set of most pathogenic variants. All resulting variants were inspected visually by unpublished genome analysis tool Rikurator (R. Katainen, V. Mäkinen, L.A. Aaltonen, E. Pitkänen, manuscript under preparation). Variants with mis-alignment or having reads only in single direction were regarded as unreliable and removed. Variant genes were annotated with Gene Ontology terms [21] and pathway analyses were done with DAVID Bioinformatics Resources 6.7 [22].

**Table 1:** Tumor cell content of pseudomyxoma peritonei FFPE samples estimated from HE stainings, and variant allele frequencies (VAF) of *KRAS* and *GNAS* from targeted sequencing.

			VAF from targeted seq*		
Patient	Grade	HE T%	*KRAS*	*GNAS*	Seq T%**
2	LG	40	0.179	0.077	16
201	LG	20	0.083	0.062	12
202	HG	10	0.054		10
206	HG	60	0.103		20
210	LG	20	0.034		6
214	LG	25	0.041	0.034	6
28	HG	50	0.078	0.079	16
29	LG	30	0.144	0.278	28
31	LG	20	0.081		16

LG: low-grade; HG: high-grade; HE: hematoxylin and eosin; T%: tumor cell percentage; VAF: variant allele frequency; seq: sequencing

* Ref. [9]

**Tumor cell percentage calculated by multiplying the heterozygote VAF (lower value of either KRAS or GNAS) by factor of two.

### Amplicon sequencing

Ultra-deep amplicon sequencing was used to validate exome sequencing results. Ninety-four most relevant variants detected with exome sequencing were validated using ultra-deep amplicon sequencing (S1 Table). We selected variants that belong to the protein kinase A (PKA) or transforming growth factor beta (TGF-β) pathway, and sites in genes which were mutated in at least two patients. Variants on PKA pathway, which is shown to play a key role in PMP pathogenesis [7], were selected since 4/9 PMP tumors lacked *GNAS* mutation and we hypothesized that in these cases mutations in other genes in the PKA pathway may lead to similar phenotype. Variants on TGF-β pathway were selected since it showed high enrichment in pathway analysis (Bonferroni corrected *p*-value 0.001; S2 Table). MAPK and ErbB pathways showed the highest enrichment of signaling pathways (Bonferroni corrected p-values 1.53x10^-6^ and 3.89x10^-5^, respectively), but these pathways were not validated due to the fact that all PMP tumor samples had an activating mutation of the *KRAS* oncogene [9], which is sufficient to activate these pathways [23]. Sample 29 could not be validated due to lack of DNA. PCR primers (Sigma-Aldrich, St. Louis, MO; S3 Table) were designed by Primer3 [24] with amplicon size of 150–200 bp, GC percentage 30–70%, primer length 18–35 bp, and melting temperature 60–62°C. PCR reactions were done in 20 μl reaction volume containing 20 ng gDNA, 0.4 μl dNTP mix (100 mM), 2 μl of each primers (10 μM), 2.5 U Expand High Fidelity^PLUS^ Enzyme Blend and 1X Expand High Fidelity^PLUS^ Reaction buffer with final concentration of 1.5 mM MgCl_2_ (Roche, Basel, Switzerland). The cycling conditions were as previously reported [25] except that denaturation time in each cycle was 30 seconds, annealing temperature was 60°C, and the number of PCR cycles was 32–35. Half of the PCR reaction was then analyzed with gel electrophoresis and the other half was used for amplicon purification. Each separate amplicon was purified with Beckman Coulter AMPure XP Beads (Pasadena, CA) according to the manual with bead:DNA ratio of 2X (20 μl of nuclease free water and 60 μl of AMpureXP Beads were added to 10 μl of sample). Purified amplicons were quantified using Qubit and all the amplicons of each patient sample were pooled in equimolar basis. The concentrations of all the amplicon pools were then adjusted to 0,54 ng/μl and 10 μl of these pools were used for library preparation. Library preparation was done using ThruPLEX DNA-seq Kit (Rubicon Genomics, Ann Arbor, MI) according to the instruction manual and libraries were checked with Agilent 2100 Bioanalyzer. Finally, the samples were sequenced with MiSeq v2 (Illumina) using paired-end sequencing with 150 base pair read length. Reads were aligned by BWA mem with default parameters and human genome build 37 (hg19) as the reference. Variants were calculated by SAMtools mpileup (v0.1.19) and VarScan (v.2.3.6) readcounts commands. Variants with coverage greater than 1,000X and frequency at least one third of the corresponding *KRAS* VAF in the same sample were regarded as confident.

### Comparison of *KRAS* and *GNAS* mutations detected with targeted sequencing, exome sequencing, and validation sequencing

Average coverage of the exome sequencing was 120X, and on average 95.9% of the target was covered with at least 20X. With this coverage, the two applied variant caller algorithms were not able to detect all the somatic mutations that we previously detected in the same samples by the targeted sequencing with the average coverage of 3,580X [9]. For *KRAS* we detected 5/9, and for *GNAS* 5/5 of the mutations (Table 2). To validate the exome sequencing results we used ultra-deep amplicon sequencing with average coverage ranging from 60,000X to 400,000X per sample. With this validation sequencing we were able to detect the *KRAS* and *GNAS* variants with similar VAF as previously with the targeted sequencing (Table 2).

**Table 2:** Comparison of *KRAS* and *GNAS* mutations detected with targeted sequencing, exome sequencing and validation sequencing.

	*KRAS*									
		Targeted sequencing*			Exome sequencing			Validation sequencing		
Patient	Position	Coverage	Call	Frequency	Coverage	Call	Frequency	Coverage	Call	Frequency
2	12:25398284	2346	C/T	0.179	29	C/T	0.207	56166	C/T	0.163
201	12:25398284	3775	C/T	0.083	54	ND	ND	310144	C/T	0.050
202	12:25398284	4159	C/T	0.054	55	C/T	0.073	415544	C/T	0.052
206	12:25398284	3640	C/T	0.103	48	ND	ND	84351	C/T	0.096
210	12:25398284	8797	C/T	0.034	48	C/T	0.083	160119	C/T	0.033
214	12:25398284	2844	C/T	0.041	56	ND	ND	165996	C/T	0.035
28	12:25398284	3495	C/T	0.078	49	C/T	0.061	259844	C/T	0.080
29	12:25398281	2765	C/T	0.144	47	C/T	0.085	NA	NA	NA
31	12:25398284	1611	C/T	0.081	28	ND	ND	25378	C/T	0.085
	*GNAS*									
		Targeted sequencing*			Exome sequencing			Validation sequencing		
Patient	Position	Coverage	Call	Frequency	Coverage	Call	Frequency	Coverage	Call	Frequency
2	20:57484420	2811	C/T	0.077	116	C/T	0.060	61926	C/T	0.094
201	20:57484421	3405	G/A	0.062	117	G/A	0.103	368512	G/A	0.048
202										
206										
210										
214	20:57484420	2509	G/A	0.034	153	G/A	0.033	265484	G/A	0.031
28	20:57484420	2726	G/A	0.079	139	G/A	0.094	164773	G/A	0.082
29	20:57484420	1763	G/A	0.278	134	G/A	0.164	NA	NA	NA
31										

* [9]; ND: not detected; NA: not available.

## Results

### Somatic exome-wide SNVs and short indels in PMP tumors

**Fig 2:**
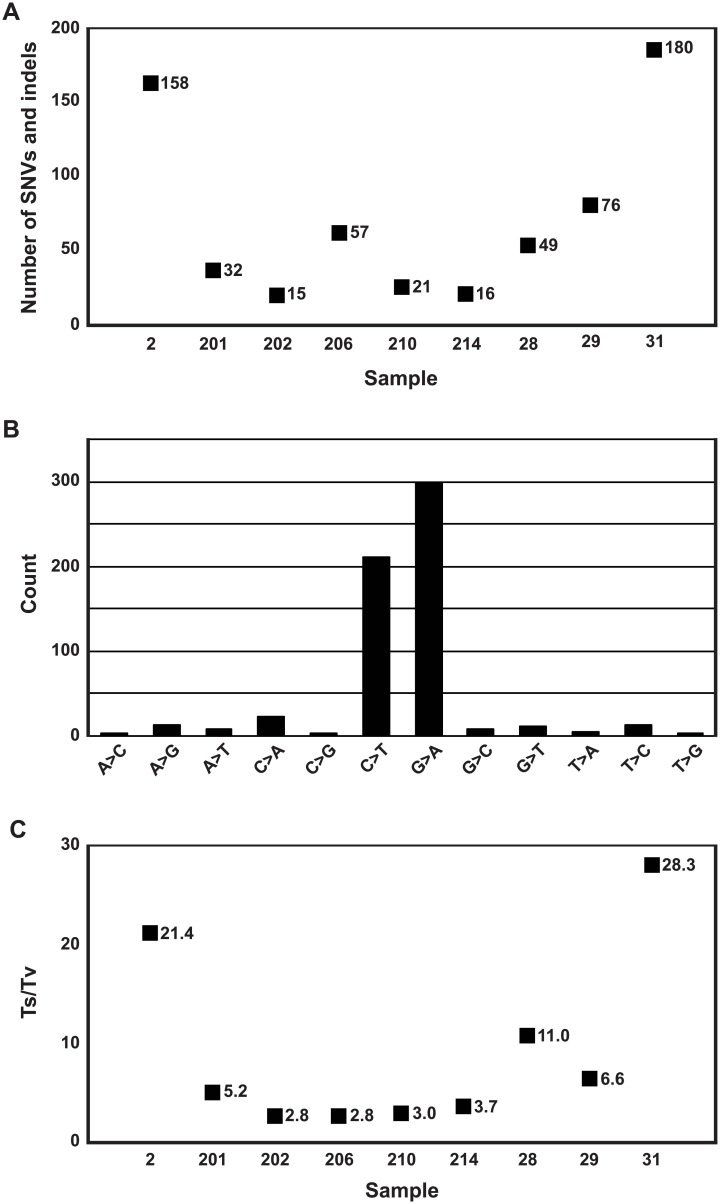
Exome-wide statistics of filtered somatic SNV and indel calls. (A) Number of SNVs and indels. (B) Total number of somatic nucleotide base changes. (C) Ts to Tv ratio of each tumor. SNV: single nucleotide variation; indel: insertion/deletion; Ts: transition; Tv: transversion.

Results of exome-wide analysis of the filtered somatic SNVs and indels are summarized in Fig 2. After all the filtering steps, we retained an average 67 high confidence, protein chancing somatic SNVs and indels per tumor (range 15–180) (S2 Table). In total, we found 535 transitions (Ts; A↔G or C↔T) and 59 transversions (Tv; A:G↔C:T), giving an overall Ts to Tv ratio 9.1. Ts to Tv ratios for each tumor ranged from 2.8 to 28.3. High rate of Ts is a common feature in FFPE samples caused by PCR induced errors, promoted by DNA modifications [26, 27].

### Validation of the mutations

We performed ultra-deep sequencing validation for altogether 94 potentially relevant variants in 53 genes detected in the exome sequencing experiment. As internal controls, we included amplicons for the *KRAS* and *GNAS* hotspot areas. Since all the sequenced samples had a *KRAS* mutation, we filtered our validation results using the corresponding *KRAS* variant allele frequencies (S1 Table). Altogether 14 mutations in 13 genes passed all the quality filtering steps, resulting in validation rate of 14.9%. All validated 14 variants were considered to affect protein function as ten of them are missense mutations, three are nonsense mutations, and one is a canonical splice site mutation. All of these mutations were predicted to be potentially functional by the *in silico* prediction tool CADD and six of them exist in the COSMIC database [28] (Table 3; the gene, transcript, and chromosomal positions are from Ensembl build 37 [29]). Evaluation of the reliability of these variants, considering the bias in FFPE samples, was accomplished by applying six separate criteria specified in the Table 3.

**Table 3:** Variants detected on pseudomyxoma peritonei by exome sequencing and validated by ultra-deep amplicon sequencing.

Patient	Gene	Ensembl gene	Ensembl transcript	Chromosomal position	Nucleotide (cDNA)	Amino acid (protein)	CADD score	Evidence*	Criteria for annotating variant as a potential artefact**
2	*OXTR*	ENSG00000180914	ENST00000316793	3:8809260	c.C614G	p.T205R	19.8		2, 3, 4
201	*LTBP1*	ENSG00000049323	ENST00000354476	2:33335707	c.G922A	p.V308M	11.3	Cosmic	1, 2, 3, 5
202	*THBS1*	ENSG00000137801	ENST00000260356	15:39885810	c.A3208G	p.T1070A	24.3		3, 4
206	*DUSP22*	ENSG00000112679	ENST00000344450	6:348195	c.G356A	p.R119H	34		1, 2, 3, 4
206	*EDNRA*	ENSG00000151617	ENST00000324300	4:148441016	c.G434A	p.R145H	23.8		1, 3, 4
206	*PRKAR1A*	ENSG00000108946	ENST00000589228	17:66518896	c.178-1G>C	splice site variant	24.7		1, 3, 4, 6
206	*RYR2*	ENSG00000198626	ENST00000366574	1:237693756	c.G2852T	p.G951V	23		3, 4
210	*TGFBR2*	ENSG00000163513	ENST00000295754	3:30729968	c.C1489T	p.R497X	50	Cosmic	
210	*PPP2R1B*	ENSG00000137713	ENST00000527614	11:111626081	c.C781T	p.R261X	38		1, 3, 4
210	*ACVR1B*	ENSG00000135503	ENST00000257963	12:52374813	c.G641T	p.G214V	31	Cosmic	3
210	*GUSB*	ENSG00000169919	ENST00000304895	7:65439369	c.G1304A	p.R435H	26.9		1, 3, 4
214	*TGFBR2*	ENSG00000163513	ENST00000295754	3:30732957	c.G1570A	p.D524N	35	Cosmic	1, 6
31	*GRIA2*	ENSG00000120251	ENST00000296526	4:158233974	c.C613T	p.R205W	28	Cosmic	1, 3, 6
31	*CNGB1*	ENSG00000070729	ENST00000251102	16:57950065	c.C2185T	p.R729X	17.9	Cosmic	1, 3

X: stop-gain. CADD score reflects the deleteriousness of the mutation.

*Cosmic: Occurence of similar mutation in the COSMIC database [28].

** Criteria for annotating a variant as a potential artefact are:
C→T/G→A reported only by one variant caller VAF < average in a sample found only in a single sample not reported as recurrently mutated in COSMIC CADD score < 15 coverage < 50% of sample average

### Validation confirmed mutations in the PKA and TGF-β pathways

**Fig 3:**
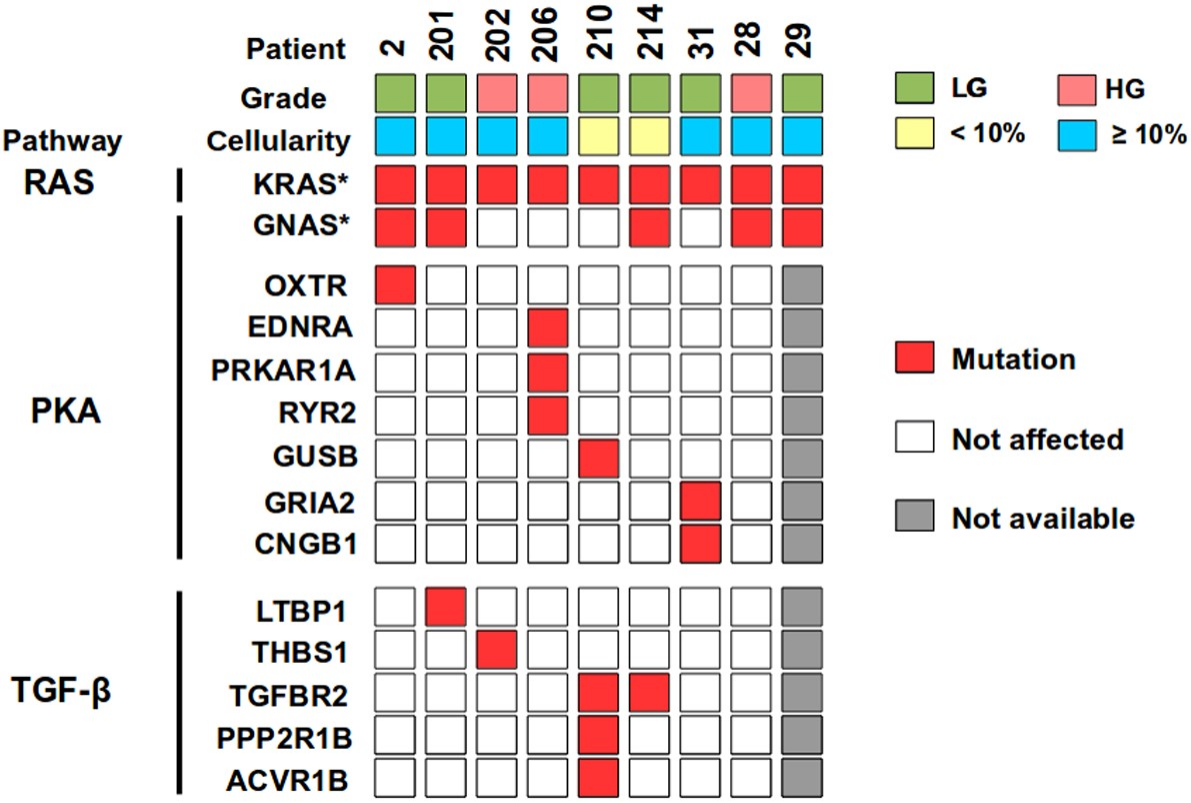
Gene mutations identified in pseudomyxoma peritonei (PMP). Nine appendix-derived PMP samples were analyzed for grade and cellularity, using HE slides and NGS, respectively. Somatic mutations were identified using exome sequencing and validated by ultra-deep amplicon sequencing. HG: high-grade; LG: low-grade. * [9].

From the selected 21 variants in 19 genes that belong to the PKA pathway, the validation confirmed seven variants in seven genes occurring in 4/9 patients (*OXTR*, *EDNRA*, *PRKAR1A*, *RYR2*, *GUSB*, *GRIA2*, and *CNGB1*) (Table 3 and Fig 3). From the selected 19 variants in 14 genes that belong to the TGF-β pathway, validation confirmed six variants in five genes occurring in 4/9 patients (*LTBP1*, *THBS1*, *TGFBR2*, *PPP2R1B*, and *ACVR1B*) (Table 3 and Fig 3). Each of these genes was mutated exclusively in one patient except *TGFBR2* which was found to be mutated in two cases.

### Other validated mutations

Finally, we selected 25 genes that were mutated in at least two PMP patients in the exome data. This led to validation sequencing of 52 variants in 25 genes. *TGFBR2* was the only mutated gene that was confirmed in multiple PMP patients (2/9). Interestingly, a mutation in *DUSP22* was confirmed in one HG PMP patient (Table 3). *DUSP22* activates the JNK pathway [30] and is involved in apoptosis. Thus, mis-regulation in apoptosis may contribute to the high-cellularity of this PMP tumor with 20% tumor cell percentage.

## Discussion

The genetic background of PMP is incompletely characterized. It is known that the RAS signaling pathway activation is frequently if not always present in PMP and most often facilitated by a mutation of the *KRAS* oncogene in codons 12 or 13 (S4 Table) [6–11,31–34]. In addition to *KRAS*, one *NRAS* mutation has been reported in a patient lacking *KRAS* mutation [10], emphasizing the crucial role of RAS pathway activation in PMP. These mutant RAS proteins are constitutively active promoting cell proliferation and suppressing apoptosis [35]. *KRAS* and *NRAS* mutated colorectal cancers (CRC) are known to be resistant to epithelial growth factor receptor (EGFR)-targeted therapy [36], suggesting that this type of treatment may also be ineffective in PMP. In addition, *BRAF* mutations are rare events in PMP [7–11] and thus anti-BRAF therapy may also not be applicable. In addition to RAS pathway activating mutations, *GNAS* codon 201 is mutated with high frequency in PMP [6–11,34]. *GNAS* encodes the α-subunit of the stimulatory G protein that transduces signals from seven-transmembrane receptors. A mutation at codon 201 leads to constitutive activation of adenylyl cyclase leading to elevated levels of cyclic AMP (cAMP) and activation of the PKA pathway. In a CRC cell line, activated GNAS has been shown to induce expression of *MUC2* and *MUC5AC* [34], which belong along with MUC5B to the most prominent mucins produced by PMP tumors [37,38]. Further, activated PKA has been shown to stimulate secretion of mucins in human colonic epithelial cells [39]. Thus, GNAS or PKA inhibition might reduce mucin secretion in PMP patients, relieving obstruction related symptoms and disease progression.

**Fig 4:**
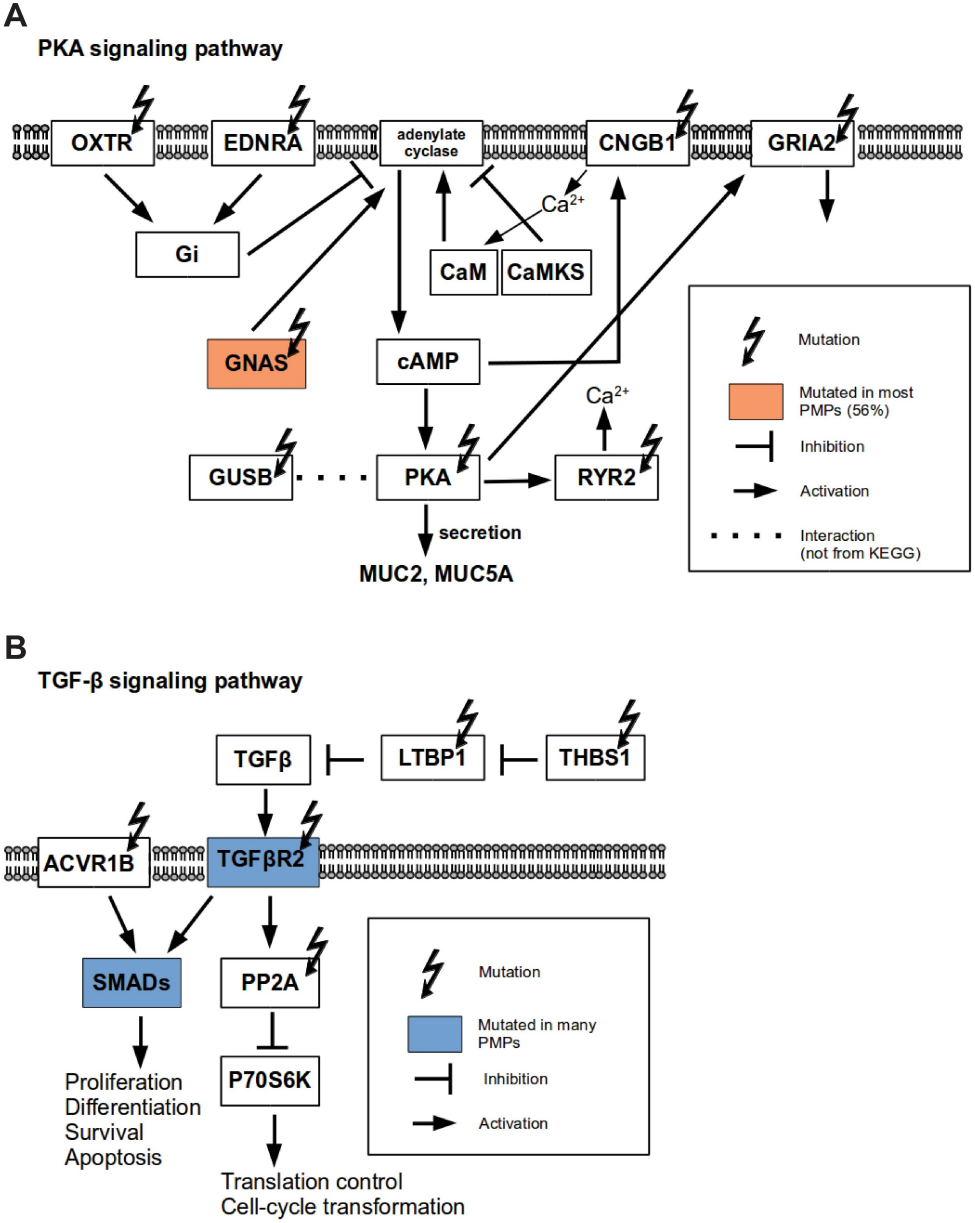
Gene mutations related to PKA and TGF-β pathways in pseudomyxoma peritonei. (A) Variants of PKA pathway. (B) Variants of TGF-β pathway. Pathways have been modified from KEGG [40]. PKA: protein kinase A; TGF-β: transforming growth factor beta.

PKA has been reported to be strongly phosphorylated and activated in PMP tumors even in the absence of *GNAS* mutation [7], which lead to the hypothesis that in addition to *GNAS*, also other members of PKA pathway may be affected. As four out of nine of our exome sequenced samples lacked the *GNAS* hotspot mutation (Fig 3), we targeted our search to identify other mutations of the PKA pathway (Fig 4A). One of our HG PMP patients harbored three mutated genes of this pathway: *PRKAR1A*, *EDNRA*, and *RYR2*. *PRKAR1A* encodes regulatory subunit of PKA and had a canonical splice site mutation (c.178-1G>C) in the dimerization/docking domain, suggesting a loss of function. Similarly, Alakus et al. [7] found a mutation in another subunit of PKA (*PRKACA*) in a PMP patient lacking *GNAS* mutation. Interestingly, patients of Carney complex, which is an autosomal dominant disease caused by inactivation of the *PRKAR1A* gene, have a high prevalence of pancreatic tumors, including intraductal papillary mucinous neoplasms (IPMN) [41]. This also supports functionality of *PRKAR1A* in PMP, since mutational profiles of IPMN and PMP are highly similar [7,9,34]. Importantly, inactivation of *PRKAR1A* gene in Carney complex cells has been found to constitutively activate PKA [42,43]. In our series, mutations in the *EDNRA* (p.R145H) and *RYR2* (p.G951V) genes were missense, altering the amino acid structure of seven-transmembrane receptor and RyR domain, respectively. One LG PMP patient had a missense mutation in the *GUSB* (p.R435H) gene, which is known to interact with the *PRKACA* subunit of PKA [44]. Furthermore, germline mutations of *GUSB* gene cause autosomal recessive disorder, mucopolysaccharidoses, characterized with production of excessive mucus. Another LG PMP patient had two mutated genes of the PKA pathway, *CNGB1* (p.R729X) and *GRIA2* (p.R205W), harboring a nonsense and a missense mutations, respectively. In summary, we were able to detect mutations in PKA pathway in 3/4 patients lacking GNAS mutation. Altogether, 8/9 patients seemed to have affected PKA pathway, making it a potentially interesting molecular target in PMP.

TGF-β pathway is dysregulated in many cancers, including PMP (S4 Table) [7,9–11]. Thus far, *SMAD4*, *SMAD2*, *SMAD3*, *TGFBR1*, and *TGFBR2* mutations have been the most often detected ones in PMP. Similarly to Alakus et al. [7], we detected *TGFBR2* mutations (p.R497X and p.D524N) in two PMP patients, both affecting kinase domains. Since *TGFBR2*-R497X is a nonsense mutation and mutations in the same residue as *TGFBR2*-D524N have been detected in inherited diseases [45], it indicates loss of function for both cases. *TGFBR2* has been found mutated also in CRC leading to inactivation of the TGF-β pathway [46]. Interestingly, only the combination of *KRAS* activation and *TGFBR2* inactivation in mice have been found to induce formation of intestinal neoplasms [47]. In addition, we detected other mutations in the TGF-β pathway: *LTBP1* (p.V308M), *THBS1* (p.T1070A), *PPP2R1B* (p.R261X), and *ACVR1B* (p.G214V) genes, which may contribute to the development and progression of PMP (Fig 4B). Both LTBP1 and THBS1 have been shown to control the activity of TGFB1 [48,49] and they were mutated in different patients. *PPP2R1B* encodes the beta isoform of the A subunit of the serine/threonine protein phosphatase 2A (*PP2A*), which has been found to be mutated also in colon and lung cancers [50]. *ACVR1B* mutation in the catalytic domain was found in a patient who also had mutated *TGFBR2*. *ACVR1B* encodes activin A type IB receptor and is frequently mutated in CRC [46]. Interestingly, loss of *ACVR1B* was recently reported to accelerate development of IPMN in mice with activated *KRAS* [51]. Altogether, 4/9 PMP patients were identified to have TGF-β pathway alterations, which further highlight the importance of the TGF-β pathway in PMP pathogenesis.

Most likely also other dysregulated pathways than RAS, PKA and TGF-β have a role in the development and progression of PMP, and further investigation of these pathways is needed (S2 and S4 Tables). For example, *TP53* has been shown to be mutated in HG PMPs [7–11]. The PI3K-AKT pathway is affected through *PIK3CA* and *AKT1* mutations [9–11], and *MCL1* amplification [6], and the Wnt pathway through *CTNNB1* mutation [7,11] and amplification of *JUN* [6]. Furthermore, mechanisms, such as somatic structural rearrangements, intronic variants, microRNA expression changes, and epigenetic events, may also influence the development of PMP but have not yet been examined.

Due to the low proportion of neoplastic cells in the PMP samples, sensitivity and functional prioritizing was emphasized in the exome sequencing data analysis. Ultra-deep targeted sequencing was then used to eliminate false positives resulting from the reduced specificity. Transition substitutions, C→T or G→A, are the most abundant mutations in PMP [7], which was evident also in our results (Fig 2B), suggesting similar mechanisms of DNA mutagenesis as in CRC [52]. It is, however, important to note that hydrolytic deamination of cytosines leads to overrepresentation of C to T (C→T/G→A), and therefore variant artefacts are common in next-generation amplicon sequencing of formalin-fixed tumor samples [27]. Prevalence of C to T artefacts is indeed highest in low concentration samples in VAFs < 10%. Although such variants are rarely of high functional relevance and do not usually locate in mutational hotspots [53], some clinically actionable mutations have been shown to be false positives [54,55]. To minimize the number of formalin-fixation caused false positive alterations in the final results, we applied the filtering steps represented in the Fig 1. In total, 14 variants (Table 3) survived after filtering and included 64% of C→T/G→A transitions (9 out of 14 substitutions), which is a higher proportion compared to the average of 40.8% in the human genome [53]. It is, however, extremely unlikely that identical artifacts emerge and pass all our filtering steps in two independent experiments utilizing distinct targeting methods, i.e. Agilent’s whole-exome hybrid capture assay for mutation identification and custom-designed PCR amplicons for ultra-deep validation sequencing. However, mutations which resemble typical artefacts in formalin-fixed tumor sequences must always be treated with a caution (Table 3).

In conclusion, we have used FFPE samples with deep sequencing to characterize the mutational landscape in PMP. FFPE samples are more abundant and easier to access than fresh samples but their computational analysis is challenging. Thus, the successful use of the freely available computational pipeline developed herein paves way to use FFPE samples in future PMP studies. Our major finding include novel mutated genes in the PKA pathway possibly contributing to the characteristic mucin production of the PMP disease. Inhibition of the PKA pathway could potentially be a universal target in reducing mucin production in the PMP patients, which could prolong survival of these patients. Our results also highlight the importance of TGF-β pathway in the development and progression of PMP and therapies targeting this pathway could also suppress progression of the disease.

## Conclusions

Genetic background of pseudomyxoma peritonei (PMP) is not well characterized and there are no targeted therapies for the treatment of this disease. In this study, we used exome sequencing to expand the mutational analysis of PMP tumors beyond the mutational hotspot areas of known cancer genes and validated the results with an ultra-deep amplicon sequencing method. Validated results indicate that in addition to the fundamental *KRAS* and *GNAS* mutations, somatic mutations in the PKA and TGF-β pathways possibly account for the increased mucus secretion and cell proliferation, respectively.

## Supporting information

S1 Table Validation amplicon sequencing results for each pseudomyxoma peritonei patient. (XLSX)

S2 Table Exome sequencing results for each pseudomyxoma peritonei patient with pathway analysis results. (XLSX)

S3 Table PCR primers used for validation amplicon sequencing. (DOCX)

S4 Table Mutation profile summary of published pseudomyxoma peritonei next-generation sequencing (NGS) analyses. (XLSX)
