# Supplementary material for: Multiple components of PKA and TGF-β pathways are mutated in pseudomyxoma peritonei
Source: PLoS One. 2017 Apr 20;12(4):e0174898. doi: 10.1371/journal.pone.0174898 (PMC5398530; doi:10.1371/journal.pone.0174898)
Supplement: S3 Table — (DOCX) [file pone.0174898.s003.docx]

**S3 Table.** PCR primers used for validation amplicon sequencing.

PRIMER PICKING RESULTS FOR ENSG00000173517_15:77471653 --> 206_PEAK1 and 2_PEAK1

OLIGO start len tm gc% any_th 3'_th hairpin seq

LEFT PRIMER 87 22 59.90 50.00 0.00 0.00 0.00 CACAGACAGTCCTACCACCAAA

RIGHT PRIMER 264 21 59.46 52.38 0.00 0.00 0.00 CCAGTTGGTGAAATGCCTCTG

PRODUCT SIZE: 178

PRIMER PICKING RESULTS FOR ENSG00000198796_18:56246157 --> 206_ALPK2

OLIGO start len tm gc% any_th 3'_th hairpin seq

LEFT PRIMER 35 22 59.70 45.45 0.00 0.00 0.00 TTCATCTCTGCTCTGCCAAAGA

RIGHT PRIMER 234 22 60.09 45.45 0.00 0.00 0.00 TGTGTTGCCTTCTTTGGAGACT

PRODUCT SIZE: 200

PRIMER PICKING RESULTS FOR ENSG00000100393_22:41545987 --> 206_EP300

OLIGO start len tm gc% any_th 3'_th hairpin seq

LEFT PRIMER 78 22 60.68 50.00 0.00 0.00 0.00 TTCATCAGAATTCACCCTCGCC

RIGHT PRIMER 275 22 59.90 50.00 0.00 0.00 0.00 GTTGTGTTGTTGGTGGTGTAGG

PRODUCT SIZE: 198

PRIMER PICKING RESULTS FOR ENSG00000112679_6:348195 --> 206_DUSP22 and 29_DUSP22

OLIGO start len tm gc% any_th 3'_th hairpin seq

LEFT PRIMER 133 22 59.83 45.45 7.78 0.00 0.00 ACACTGGTGATCGCATACATCA

RIGHT PRIMER 305 22 59.50 50.00 0.00 0.00 0.00 TCTGATGTCCCCTAAGAACTGC

PRODUCT SIZE: 173

PRIMER PICKING RESULTS FOR ENSG00000151617_4:148441016 --> 206_EDNRA

OLIGO start len tm gc% any_th 3'_th hairpin seq

LEFT PRIMER 118 22 59.96 45.45 0.00 0.00 0.00 AAAACTGTAAGTGCCCACATGC

RIGHT PRIMER 317 22 60.35 50.00 0.00 0.00 0.00 TACCTGTCAACACTAAGAGCGC

PRODUCT SIZE: 200

PRIMER PICKING RESULTS FOR ENSG00000108946_17:66518896 --> 206_PRKAR1A

OLIGO start len tm gc% any_th 3'_th hairpin seq

LEFT PRIMER 138 22 59.96 45.45 0.00 0.00 0.00 ACATGAGAGTGCCAGCTTTACA

RIGHT PRIMER 309 22 60.36 54.55 0.00 0.00 0.00 GCCTCCTACCTTTAACCACTGG

PRODUCT SIZE: 172

PRIMER PICKING RESULTS FOR ENSG00000198626_1:237693756 --> 206_RYR2

OLIGO start len tm gc% any_th 3'_th hairpin seq

LEFT PRIMER 167 22 59.51 50.00 0.00 0.00 0.00 CCAGGACTTTGTTGGCATTAGG

RIGHT PRIMER 346 22 59.96 45.45 0.00 0.00 0.00 AAAAGCAAGGCATCTGTGTGTC

PRODUCT SIZE: 180

PRIMER PICKING RESULTS FOR 206_ENSG00000066455_14:93264147 --> 206_GOLGA5

OLIGO start len tm gc% any_th 3'_th hairpin seq

LEFT PRIMER 89 25 62.10 48.00 0.00 0.00 0.00 AAGTAGGATCTCGGACACCAGTAGA

RIGHT PRIMER 241 25 62.13 48.00 2.30 0.00 0.00 CGGTAGGCTCCTTCTGTGAACTATT

PRODUCT SIZE: 153

PRIMER PICKING RESULTS FOR 206_ENSG00000110619_11:3039154 --> 206_CARS

OLIGO start len tm gc% any_th 3'_th hairpin seq

LEFT PRIMER 89 26 60.41 38.46 0.00 0.00 0.00 TCACAGCAGTAGAAAGTTGTAACAGA

RIGHT PRIMER 247 25 62.20 48.00 0.00 0.00 0.00 TCTTCTCCCCACTTCTCAAACTGAC

PRODUCT SIZE: 159

PRIMER PICKING RESULTS FOR 206_ENSG00000118503_6:138200305 --> 206_TNFAIP3

OLIGO start len tm gc% any_th 3'_th hairpin seq

LEFT PRIMER 153 24 63.67 54.17 0.00 0.00 0.00 GTCACCAGCGTTCCAAGTCAGATC

RIGHT PRIMER 348 25 62.69 48.00 0.73 0.73 0.00 CAAAAGCCCTTGTTTTCTGGAGTCC

PRODUCT SIZE: 196

PRIMER PICKING RESULTS FOR 206_ENSG00000127914_7:91603084 --> 206_AKAP9

OLIGO start len tm gc% any_th 3'_th hairpin seq

LEFT PRIMER 52 25 61.98 44.00 0.00 0.00 0.00 TAAAAGAGAGACTCCCAAAGCTGCT

RIGHT PRIMER 239 25 60.56 40.00 0.00 0.00 0.00 CACATCATGTTTACTGCTTGACGTT

PRODUCT SIZE: 188

PRIMER PICKING RESULTS FOR 29_ENSG00000173517_15:77471978 --> 29_PEAK1

OLIGO start len tm gc% any_th 3'_th hairpin seq

LEFT PRIMER 97 23 62.32 56.52 0.00 0.00 0.00 CTCCCCAGCAAAGATCCAGAGAG

RIGHT PRIMER 269 25 62.03 48.00 0.00 0.00 0.00 CAGATTGAGGTGTTTCTGGAGGAGA

PRODUCT SIZE: 173

PRIMER PICKING RESULTS FOR 29_ENSG00000175054_3:142168390 --> 29_ATR

OLIGO start len tm gc% any_th 3'_th hairpin seq

LEFT PRIMER 141 24 60.69 41.67 1.77 1.77 0.00 TTTTAGGCCAAGACCCATGTTCTT

RIGHT PRIMER 290 28 61.31 39.29 0.00 0.00 0.00 CTGGCATAGTAAGTTTTCATCAGTAGCT

PRODUCT SIZE: 150

PRIMER PICKING RESULTS FOR 29_ENSG00000140443_15:99465416 --> 29_IGF1R

OLIGO start len tm gc% any_th 3'_th hairpin seq

LEFT PRIMER 133 25 61.50 44.00 0.00 0.00 0.00 AAAAGCCACATTTCTCTCCTCCTTG

RIGHT PRIMER 317 25 61.85 48.00 0.00 0.00 0.00 CTCCTTGTTATCCACTCTGCTCTCA

PRODUCT SIZE: 185

PRIMER PICKING RESULTS FOR 29_ENSG00000141646_18:48581262 --> 29_SMAD4

OLIGO start len tm gc% any_th 3'_th hairpin seq

LEFT PRIMER 104 25 60.63 40.00 0.00 0.00 0.00 TGGTGAAGGATGAATATGTGCATGA

RIGHT PRIMER 258 25 62.20 48.00 0.00 0.00 0.00 AGCATTAGACTCAGATGGGGCTAAC

PRODUCT SIZE: 155

PRIMER PICKING RESULTS FOR 29_ENSG00000181555_3:47164642 --> 29_SETD2

OLIGO start len tm gc% any_th 3'_th hairpin seq

LEFT PRIMER 56 25 61.95 48.00 0.00 0.00 0.00 TCTCGGCCATACACAGATAACAGAG

RIGHT PRIMER 245 27 61.90 40.74 0.00 0.00 0.00 ACTTGCCTCTTCTTTCCATCTCTAAGT

PRODUCT SIZE: 190

PRIMER PICKING RESULTS FOR 29_ENSG00000169679_2:111413445 --> 29_BUB1

OLIGO start len tm gc% any_th 3'_th hairpin seq

LEFT PRIMER 98 25 61.35 44.00 0.00 0.00 0.00 TCATGGACTAAAGAGGGAGATTGCT

RIGHT PRIMER 259 25 62.41 52.00 0.00 0.00 0.00 GATGTGAAGTCTCCTGGGCTCTTAG

PRODUCT SIZE: 162

PRIMER PICKING RESULTS FOR ENSG00000067900_18:18690964 --> 206_ROCK1

OLIGO start len tm gc% any_th 3'_th hairpin seq

LEFT PRIMER 57 18 60.05 61.11 0.00 0.00 0.00 CGGGTGGGATGCGACTTT

RIGHT PRIMER 274 20 61.84 60.00 0.00 0.00 0.00 TGCCCTCTTACCAGCACCAG

PRODUCT SIZE: 218

GNAS

OLIGO start len tm gc% any_th 3'_th hairpin seq

LEFT PRIMER 131 25 62.46 44.00 0.00 0.00 0.00 ATTACTGTTTCGGTTGGCTTTGGTG

RIGHT PRIMER 317 25 62.29 48.00 1.30 0.00 0.00 CTGGGGTGAATGTCAAGAAACCATG

PRODUCT SIZE: 187

PRIMER PICKING RESULTS FOR 202_ENSG00000029363_6:136589425 ---> 202_BCLAF1

OLIGO start len tm gc% any_th 3'_th hairpin seq

LEFT PRIMER 78 25 61.75 44.00 8.08 0.00 0.00 TCTGTTGTAAGATTCAGCAGGGTCT

RIGHT PRIMER 244 27 61.18 40.74 0.00 0.00 0.00 TCCTTCTTACTCTCCTTTTCTTCTCGA

PRODUCT SIZE: 167

PRIMER PICKING RESULTS FOR 202_ENSG00000135973_2:105859066 ---> 202_GPR45

OLIGO start len tm gc% any_th 3'_th hairpin seq

LEFT PRIMER 91 25 63.83 52.00 0.00 0.00 0.00 CTACATGTGCATCCTCAACACGGTC

RIGHT PRIMER 268 25 62.65 52.00 0.00 0.00 0.00 GAAGAGGATCAGGATGGTGGTGAAG

PRODUCT SIZE: 178

PRIMER PICKING RESULTS FOR 202_ENSG00000137801_15:39885810 ---> 202_THBS1

OLIGO start len tm gc% any_th 3'_th hairpin seq

LEFT PRIMER 100 24 61.98 45.83 0.00 0.00 0.00 TGTTGTGATGTGGAAGCAAGTCAC

RIGHT PRIMER 299 25 62.19 52.00 0.00 0.00 0.00 CCCATAAGCTCTCTCTCTGTTCCAG

PRODUCT SIZE: 200

PRIMER PICKING RESULTS FOR 28_ENSG00000140443_15:99454613 ---> 28_IGF1R

OLIGO start len tm gc% any_th 3'_th hairpin seq

LEFT PRIMER 67 25 62.21 48.00 0.00 0.00 0.00 CAAGACAGGTGCTTTTCAGAGACAC

RIGHT PRIMER 254 25 61.96 44.00 0.00 0.00 0.00 TCCTTGTAGTAAACGGTGAAGCTGA

PRODUCT SIZE: 188

PRIMER PICKING RESULTS FOR 28_ENSG00000152217_18:42281616 ---> 28_SETBP1

OLIGO start len tm gc% any_th 3'_th hairpin seq

LEFT PRIMER 143 25 61.33 44.00 0.00 0.00 0.00 GAGATGGTTTGGAAGAGCAGGAATT

RIGHT PRIMER 294 25 61.92 48.00 11.64 0.00 0.00 GATGGTGATCTTGATCTCAGGTGGA

PRODUCT SIZE: 152

PRIMER PICKING RESULTS FOR 28_ENSG00000198752_14:103410741 ---> 28_CDC42BPB

OLIGO start len tm gc% any_th 3'_th hairpin seq

LEFT PRIMER 127 24 63.43 54.17 10.73 10.73 0.00 GTGCCGCTGACTGTAAGAAGGTAC

RIGHT PRIMER 325 22 63.11 59.09 0.00 0.00 0.00 GAGTTCCTCTTGAGTGTGGCCG

PRODUCT SIZE: 199

PRIMER PICKING RESULTS FOR 2_ENSG00000173517_15:77473476 ---> 2_PEAK1

OLIGO start len tm gc% any_th 3'_th hairpin seq

LEFT PRIMER 155 25 62.12 48.00 0.00 0.00 0.00 CGAGAGTTGGGATGAGAGTGATGAA

RIGHT PRIMER 342 25 61.86 44.00 0.00 0.00 0.00 TGTCATCATAGTCCACAGCACAGAT

PRODUCT SIZE: 188

PRIMER PICKING RESULTS FOR 2_ENSG00000175054_3:142274739 ---> 2_ATR

OLIGO start len tm gc% any_th 3'_th hairpin seq

LEFT PRIMER 160 25 62.24 44.00 0.00 0.00 0.00 TTCTGTCTGCAAGCCATTCCTTTTC

RIGHT PRIMER 350 27 60.85 37.04 0.00 0.00 0.00 ACTGAGCACTGAATCTATAAAGCATGT

PRODUCT SIZE: 191

PRIMER PICKING RESULTS FOR 2_ENSG00000057657_6:106552898 ---> 2_PRDM1_1

OLIGO start len tm gc% any_th 3'_th hairpin seq

LEFT PRIMER 149 25 62.17 44.00 0.00 0.00 0.00 TTTCACCCCTCACATCAGAAAAGGA

RIGHT PRIMER 344 24 62.98 54.17 0.00 0.00 0.00 CTTGGAGTGGTGGAGGATGGAATG

PRODUCT SIZE: 196

PRIMER PICKING RESULTS FOR 2_ENSG00000057657_6:106553275 ---> 2_PRDM1_2

OLIGO start len tm gc% any_th 3'_th hairpin seq

LEFT PRIMER 72 24 62.96 54.17 0.00 0.00 0.00 GGGACTCCTACGCTTACTTGAACG

RIGHT PRIMER 235 25 62.49 48.00 5.01 0.00 0.00 CAGGCCATTACAATTCATGCCGTAG

PRODUCT SIZE: 164

PRIMER PICKING RESULTS FOR 210_ENSG00000175054_3:142242936 --> 210_ATR

OLIGO start len tm gc% any_th 3'_th hairpin seq

LEFT PRIMER 109 25 60.92 40.00 2.61 0.00 0.00 AGTATGCAACAGACAGTGAAACAGT

RIGHT PRIMER 260 25 61.67 48.00 4.34 0.00 0.00 ATCTAATCGACCTGGATCTATCGCC

PRODUCT SIZE: 152

PRIMER PICKING RESULTS FOR 210_ENSG00000057657_6:106553776 --> 210_PRDM1

OLIGO start len tm gc% any_th 3'_th hairpin seq

LEFT PRIMER 117 25 61.77 44.00 0.00 0.00 0.00 AAAGAAACATGACCGGCTACAAGAC

RIGHT PRIMER 288 25 61.95 40.00 21.98 0.00 0.00 ATACAAACACAAGCATGCACTCACA

PRODUCT SIZE: 172

PRIMER PICKING RESULTS FOR 210_ENSG00000108821_17:48267430 --> 210_COL1A1

OLIGO start len tm gc% any_th 3'_th hairpin seq

LEFT PRIMER 113 22 62.33 59.09 0.00 0.00 0.00 GAAACTCCTGCCTCCTTCCCTC

RIGHT PRIMER 281 20 61.88 60.00 0.00 0.00 0.00 CAAGCCACTCACAATGGGGC

PRODUCT SIZE: 169

PRIMER PICKING RESULTS FOR 210_ENSG00000163513_3:30729968 --> 210_TGFBR2

OLIGO start len tm gc% any_th 3'_th hairpin seq

LEFT PRIMER 157 25 62.75 48.00 4.13 2.08 0.00 CTGTGTCGAAAGCATGAAGGACAAC

RIGHT PRIMER 336 25 62.10 48.00 0.00 0.00 0.00 GGACCTTCCCTCAGATTTAAGCTCA

PRODUCT SIZE: 180

PRIMER PICKING RESULTS FOR 210_ENSG00000137713_11:111626081 --> 210_PPP2R1B

OLIGO start len tm gc% any_th 3'_th hairpin seq

LEFT PRIMER 148 25 62.04 44.00 0.00 0.00 0.00 TTGCCCAGTTATTGTCTCAGGATGA

RIGHT PRIMER 330 25 60.75 40.00 0.00 0.00 0.00 TGGGTACAACAGCAAATCTATGTGT

PRODUCT SIZE: 183

PRIMER PICKING RESULTS FOR 210_ENSG00000135503_12:52374813 --> 210_ACVR1B

OLIGO start len tm gc% any_th 3'_th hairpin seq

LEFT PRIMER 67 25 61.96 44.00 6.16 0.00 0.00 GCCAATTAGTGTGGGAGTTGGAAAA

RIGHT PRIMER 265 25 61.84 44.00 0.00 0.00 0.00 ATATTTTCACAGCCACATCACCACC

PRODUCT SIZE: 199

PRIMER PICKING RESULTS FOR 210_ENSG00000169919_7:65439369 --> 210_GUSB

OLIGO start len tm gc% any_th 3'_th hairpin seq

LEFT PRIMER 149 25 62.43 44.00 0.00 0.00 0.00 TTCAACAACGTTTCTCTGCATCACC

RIGHT PRIMER 298 25 62.29 48.00 8.98 0.00 0.00 GAGCACTCACTTCAAGTAGTAGCCA

PRODUCT SIZE: 150

PRIMER PICKING RESULTS FOR 214_ENSG00000175054_3:142259808 --> 214_ATR

OLIGO start len tm gc% any_th 3'_th hairpin seq

LEFT PRIMER 59 25 63.15 52.00 0.00 0.00 0.00 CTTGTCCTTAGGGCTCATCTGCTTC

RIGHT PRIMER 240 25 62.88 48.00 8.43 0.53 0.00 ATCCTTGAATCGAAGGCCAGTTCTC

PRODUCT SIZE: 182

PRIMER PICKING RESULTS FOR 214_ENSG00000073331_4:113350341 --> 214_ALPK1

OLIGO start len tm gc% any_th 3'_th hairpin seq

LEFT PRIMER 136 25 61.93 44.00 0.00 0.00 0.00 TTCCACTTAGCCTCCTGTTTTCTCA

RIGHT PRIMER 333 25 63.33 52.00 1.41 0.00 0.00 GTTACATCAGAAAGCACCCTGGGAG

PRODUCT SIZE: 198

PRIMER PICKING RESULTS FOR 214_ENSG00000108821_17:48266160 --> 214_COL1A1

OLIGO start len tm gc% any_th 3'_th hairpin seq

LEFT PRIMER 80 23 62.77 56.52 0.00 0.00 0.00 GGTGAATCTGGACGTGAGGTGAG

RIGHT PRIMER 279 25 62.11 48.00 0.00 0.00 0.00 CATGGAGTGTTGCCATCTTACCTTG

PRODUCT SIZE: 200

PRIMER PICKING RESULTS FOR 214_ENSG00000116128_1:147090629 --> 214_BCL9

OLIGO start len tm gc% any_th 3'_th hairpin seq

LEFT PRIMER 44 25 61.99 44.00 2.11 0.00 0.00 CAAAAGAGGGTTGTACGTGAGCAAT

RIGHT PRIMER 240 25 62.83 48.00 0.00 0.00 0.00 TTGTGGGAGAGGTTTCGGATCATTC

PRODUCT SIZE: 197

PRIMER PICKING RESULTS FOR 214_ENSG00000163513_3:30732957 --> 214_TGFBR2

OLIGO start len tm gc% any_th 3'_th hairpin seq

LEFT PRIMER 163 25 63.49 52.00 0.00 0.00 0.00 GATGGTGTGTGAGACGTTGACTGAG

RIGHT PRIMER 333 25 61.81 44.00 0.00 0.00 0.00 TATTTGGTAGTGTTTAGGGAGCCGT

PRODUCT SIZE: 171

PRIMER PICKING RESULTS FOR 31_ENSG00000109756_4:160273905 --> 31_RAPGEF2

OLIGO start len tm gc% any_th 3'_th hairpin seq

LEFT PRIMER 120 25 61.66 44.00 0.00 0.00 0.00 TTTCTCATGCAGGCTATACTTTGGC

RIGHT PRIMER 307 25 61.88 48.00 5.07 3.02 0.00 CATCCCTAGGTTTGTTTCCACGATG

PRODUCT SIZE: 188

PRIMER PICKING RESULTS FOR 31_ENSG00000072062_19:14213651 --> 31_PRKACA

OLIGO start len tm gc% any_th 3'_th hairpin seq

LEFT PRIMER 138 25 61.77 44.00 0.00 0.00 0.00 AAACAGATCGAACACACCCTGAATG

RIGHT PRIMER 337 23 62.15 52.17 0.00 0.00 0.00 CTTCCACAAAAGAGAGCAGCCAC

PRODUCT SIZE: 200

PRIMER PICKING RESULTS FOR 31_ENSG00000173517_15:77425591 --> 31_PEAK1

OLIGO start len tm gc% any_th 3'_th hairpin seq

LEFT PRIMER 65 25 62.01 44.00 0.00 0.00 0.00 AACAGCATTTCCAGCTTAACCACTC

RIGHT PRIMER 249 25 62.04 44.00 21.42 0.00 0.00 TGAAGGCTTCGGATTTTACCCACTA

PRODUCT SIZE: 185

PRIMER PICKING RESULTS FOR 31_ENSG00000057657_6:106534460 --> 31_PRDM1

OLIGO start len tm gc% any_th 3'_th hairpin seq

LEFT PRIMER 127 25 62.19 52.00 0.00 0.00 0.00 GGAGAATGTGGACTGGGTAGAGATG

RIGHT PRIMER 326 25 61.65 44.00 0.00 0.00 0.00 CATGTTTCTACTGCGACATTAGCCa

PRODUCT SIZE: 200

PRIMER PICKING RESULTS FOR 31_ENSG00000073331_4:113353140 --> 31_ALPK1_1

OLIGO start len tm gc% any_th 3'_th hairpin seq

LEFT PRIMER 86 25 62.16 48.00 1.74 0.00 0.00 GGCCCTACATTTAAAGCTAGTCCCT

RIGHT PRIMER 236 25 62.17 48.00 0.00 0.00 0.00 AATTAGGGGTGAAGGAGCTACATGG

PRODUCT SIZE: 151

PRIMER PICKING RESULTS FOR 31_ENSG00000073331_4:113351611 --> 31_ALPK1_2 and 2_ALPK1

OLIGO start len tm gc% any_th 3'_th hairpin seq

LEFT PRIMER 131 25 61.38 40.00 0.00 0.00 0.00 TGTCCTGATTCACTTGCAGTTTTCA

RIGHT PRIMER 327 25 62.60 48.00 0.00 0.00 0.00 AACAGGCTCATCATCTCTCTTGGTG

PRODUCT SIZE: 197

PRIMER PICKING RESULTS FOR 31_ENSG00000116128_1:147087695 --> 31_BCL9

OLIGO start len tm gc% any_th 3'_th hairpin seq

LEFT PRIMER 121 25 61.90 44.00 10.21 0.00 0.00 GTTTTGAAGGGCCAGGTTGAAACTA

RIGHT PRIMER 281 25 62.20 44.00 0.00 0.00 0.00 AAATTCACCACCTCCTTTCCTTTGC

PRODUCT SIZE: 161

PRIMER PICKING RESULTS FOR 2_ENSG00000057657_6:106552783 --> 2_PRDM1_3

OLIGO start len tm gc% any_th 3'_th hairpin seq

LEFT PRIMER 120 25 61.94 44.00 0.00 0.00 0.00 AAACACAGAGCAGTCTAAAGCAACC

RIGHT PRIMER 314 25 62.11 48.00 0.00 0.00 0.00 TCCCACGTCTTCTAAAGTCATCGAG

PRODUCT SIZE: 195

PRIMER PICKING RESULTS FOR 2_ENSG00000073331_4:113298844 --> 2_ALPK1

OLIGO start len tm gc% any_th 3'_th hairpin seq

LEFT PRIMER 108 25 62.03 52.00 0.00 0.00 0.00 CCTATTATCCCTCCCTGCTTGAGTC

RIGHT PRIMER 266 25 62.21 52.00 7.94 0.00 0.00 CCCTGGGTCTAGGGTGATCAATTAC

PRODUCT SIZE: 159

PRIMER PICKING RESULTS FOR 2_ENSG00000108821_17:48265929 --> 2_COL1A1

OLIGO start len tm gc% any_th 3'_th hairpin seq

LEFT PRIMER 101 23 62.14 56.52 0.00 0.00 0.00 CTGACCCATATTCCCCTGCTCTC

RIGHT PRIMER 250 25 62.11 48.00 0.00 0.00 0.00 CAGCTACTTACAGTCTCACCACGAT

PRODUCT SIZE: 150

PRIMER PICKING RESULTS FOR 2_ENSG00000116128_1:147090856 --> 2_BCL9_1

OLIGO start len tm gc% any_th 3'_th hairpin seq

LEFT PRIMER 47 24 61.90 50.00 13.48 13.48 0.00 TCTTCCCAGAATACCAGACTGCAG

RIGHT PRIMER 245 25 63.02 48.00 0.00 0.00 0.00 CTGCCCTATTGTTGGGAGTTGAGTT

PRODUCT SIZE: 199

PRIMER PICKING RESULTS FOR 2_ENSG00000116128_1:147095690 --> 2_BCL9_2

OLIGO start len tm gc% any_th 3'_th hairpin seq

LEFT PRIMER 156 25 60.97 44.00 0.00 0.00 0.00 TGGGCATTAATACACAGAATCCTCG

RIGHT PRIMER 352 25 61.26 48.00 0.00 0.00 0.00 CCCCATGATAGGATTGTGTGACATC

PRODUCT SIZE: 197

PRIMER PICKING RESULTS FOR 2_ENSG00000140443_15:99250917 --> 2_IGF1R_1

OLIGO start len tm gc% any_th 3'_th hairpin seq

LEFT PRIMER 50 25 61.98 48.00 0.00 0.00 0.00 GTTTACCCTCTTGTCTCCCTTCAGT

RIGHT PRIMER 243 25 62.13 48.00 0.00 0.00 0.00 GAACAGCAGCAAGTACTCGGTAATG

PRODUCT SIZE: 194

PRIMER PICKING RESULTS FOR 2_ENSG00000140443_15:99460090 --> 2_IGF1R_2

OLIGO start len tm gc% any_th 3'_th hairpin seq

LEFT PRIMER 157 24 61.63 45.83 0.00 0.00 0.00 GAGGCTGAATACCGCAAAGTCTTT

RIGHT PRIMER 309 24 62.07 50.00 4.05 4.05 0.00 CTGCTGACTTACACACTACCTGGT

PRODUCT SIZE: 153

PRIMER PICKING RESULTS FOR 2_ENSG00000140443_15:99467177 --> 2_IGF1R_3

OLIGO start len tm gc% any_th 3'_th hairpin seq

LEFT PRIMER 124 25 62.60 48.00 4.61 4.61 0.00 ACAGAAGGAGCAGATGACATTCCTG

RIGHT PRIMER 317 25 61.38 44.00 0.00 0.00 0.00 TGCTGTCAATGGATGGAAGTACATG

PRODUCT SIZE: 194

PRIMER PICKING RESULTS FOR 2_ENSG00000141646_18:48604790 --> 2_SMAD4

OLIGO start len tm gc% any_th 3'_th hairpin seq

LEFT PRIMER 90 25 61.72 44.00 0.00 0.00 0.00 TCAGGATGAGTTTTGTGAAAGGCTG

RIGHT PRIMER 244 25 62.05 48.00 0.00 0.00 0.00 GTCTAAAGGTTGTGGGTCTGCAATC

PRODUCT SIZE: 155

PRIMER PICKING RESULTS FOR 2_ENSG00000152217_18:42532265 --> 2_SETBP1_1

OLIGO start len tm gc% any_th 3'_th hairpin seq

LEFT PRIMER 128 25 61.93 44.00 0.00 0.00 0.00 AGTTCCAAGTGTTCAGAATCTCCCA

RIGHT PRIMER 303 25 61.81 48.00 0.00 0.00 0.00 CCTACCACGCTTCTTCTTTGACTTC

PRODUCT SIZE: 176

PRIMER PICKING RESULTS FOR 2_ENSG00000152217_18:42532148 --> 2_SETBP1_2

OLIGO start len tm gc% any_th 3'_th hairpin seq

LEFT PRIMER 164 25 61.46 44.00 0.00 0.00 0.00 CAAAGCACAAGAGGAAACGGAAAAG

RIGHT PRIMER 313 25 61.26 44.00 0.00 0.00 0.00 AAATGCTGGGATATGGATTCTCGTG

PRODUCT SIZE: 150

PRIMER PICKING RESULTS FOR 2_ENSG00000152217_18:42531571 --> 2_SETBP1_3

OLIGO start len tm gc% any_th 3'_th hairpin seq

LEFT PRIMER 52 25 62.03 40.00 0.00 0.00 0.00 AAGCAAACTGGGCAAGCAGATTAAT

RIGHT PRIMER 239 25 62.66 48.00 9.61 9.61 0.00 CAAGTGACTGAAAGTTGGAAGGCAC

PRODUCT SIZE: 188

PRIMER PICKING RESULTS FOR 2_ENSG00000181555_3:47163199 --> 2_SETD2

OLIGO start len tm gc% any_th 3'_th hairpin seq

LEFT PRIMER 106 25 61.87 44.00 0.00 0.00 0.00 TTCCAGGGAGAACAGGCGTAATAAT

RIGHT PRIMER 255 25 61.81 44.00 0.00 0.00 0.00 TCAGAAGTATGCACATGTCCTCCTT

PRODUCT SIZE: 150

PRIMER PICKING RESULTS FOR 2_ENSG00000198752_14:103412907 --> 2_CDC42BPB and 31_CDC42BPB

OLIGO start len tm gc% any_th 3'_th hairpin seq

LEFT PRIMER 41 25 62.25 44.00 0.00 0.00 0.00 TGGCGTTTGATGACTTCTCTCTTCT

RIGHT PRIMER 240 24 63.18 50.00 0.00 0.00 0.00 GAACATGCACGACCTGATTCCTCA

PRODUCT SIZE: 200

PRIMER PICKING RESULTS FOR 2_ENSG00000198796_18:56182275 --> 2_ALPK2

OLIGO start len tm gc% any_th 3'_th hairpin seq

LEFT PRIMER 123 25 62.09 48.00 0.00 0.00 0.00 CTGTAGTCACTGGGGCTTTCTATGT

RIGHT PRIMER 276 25 62.22 48.00 0.00 0.00 0.00 CACAAGGCAGGGTAAACACACTTAC

PRODUCT SIZE: 154

PRIMER PICKING RESULTS FOR 2_ENSG00000177084_12:133257207 --> 2_POLE

OLIGO start len tm gc% any_th 3'_th hairpin seq

LEFT PRIMER 91 25 62.02 48.00 0.00 0.00 0.00 TGTAGAGGATGGTCTTGTGTCTGTG

RIGHT PRIMER 282 25 61.96 44.00 4.15 0.00 0.00 TGACAGTCACAGAGCTACATGAACA

PRODUCT SIZE: 192

PRIMER PICKING RESULTS FOR 2_ENSG00000168036_3:41266954 --> 2_CTNNB1

OLIGO start len tm gc% any_th 3'_th hairpin seq

LEFT PRIMER 87 25 62.16 48.00 14.83 6.11 0.00 CTGCAGTTATGGTCCATCAGCTTTC

RIGHT PRIMER 253 25 61.99 48.00 0.00 0.00 0.00 CTCACGATGATGGGAAAGGTTATGC

PRODUCT SIZE: 167

PRIMER PICKING RESULTS FOR 2_ENSG00000123612_2:158397620 --> 2_ACVR1C

OLIGO start len tm gc% any_th 3'_th hairpin seq

LEFT PRIMER 101 25 61.44 44.00 0.00 0.00 0.00 GGCTCCTGAAATGCTTGATGATACA

RIGHT PRIMER 260 25 61.63 40.00 0.00 0.00 0.00 TCAAGGAAAGGCAAACACAAAGGAT

PRODUCT SIZE: 160

PRIMER PICKING RESULTS FOR 31_ENSG00000152217_18:42530108 --> 31_SETBP1_1

OLIGO start len tm gc% any_th 3'_th hairpin seq

LEFT PRIMER 83 23 61.71 52.17 0.00 0.00 0.00 GACCCGTCACTCAGAATTGCTTC

RIGHT PRIMER 233 24 60.74 45.83 8.36 2.86 0.00 TTAGACAACTGACTCCACGTGTTC

PRODUCT SIZE: 151

PRIMER PICKING RESULTS FOR 31_ENSG00000152217_18:42530894 --> 31_SETBP1_2

OLIGO start len tm gc% any_th 3'_th hairpin seq

LEFT PRIMER 65 25 62.16 48.00 0.00 0.00 0.00 ATGCTGAGAAAGTTATCCCAGGAGG

RIGHT PRIMER 237 25 63.24 48.00 0.00 0.00 0.00 CATGGTGCTAGGTTTTGGTTTGCTG

PRODUCT SIZE: 173

PRIMER PICKING RESULTS FOR 31_ENSG00000181555_3:47161750 --> 31_SETD2

OLIGO start len tm gc% any_th 3'_th hairpin seq

LEFT PRIMER 78 25 62.21 44.00 0.00 0.00 0.00 ATGGTGAGCTTCAGGACAGAAAGAA

RIGHT PRIMER 246 25 59.69 36.00 0.52 0.52 0.00 TCAAAGTAACATGGCATTTTCCCTT

PRODUCT SIZE: 169

PRIMER PICKING RESULTS FOR 31_ENSG00000198752_14:103434646 --> 31_CDC42BPB

OLIGO start len tm gc% any_th 3'_th hairpin seq

LEFT PRIMER 78 25 61.56 44.00 0.00 0.00 0.00 TGATACCCACGCCATAGATACGAAT

RIGHT PRIMER 243 25 56.78 32.00 0.00 0.00 0.00 CAGTTAGCTTCTTGTTTTCATCAAA

PRODUCT SIZE: 166

PRIMER PICKING RESULTS FOR 31_ENSG00000198796_18:56203241 --> 31_ALPK2

OLIGO start len tm gc% any_th 3'_th hairpin seq

LEFT PRIMER 159 25 61.77 44.00 0.00 0.00 0.00 AACAACTCAAGATGGATCACACTGC

RIGHT PRIMER 349 25 62.57 44.00 0.00 0.00 0.00 GCCCATGTTTCCGTCATTTGATTGA

PRODUCT SIZE: 191

PRIMER PICKING RESULTS FOR 31_ENSG00000067900_18:18547714 --> 31_ROCK1_1

OLIGO start len tm gc% any_th 3'_th hairpin seq

LEFT PRIMER 157 26 61.35 46.15 0.00 0.00 0.00 CCAGATGGTAGTGAAACATCAGAAGG

RIGHT PRIMER 354 25 61.15 44.00 0.00 0.00 0.00 ACAGCCATATCCCAATTTCAGTCTG

PRODUCT SIZE: 198

PRIMER PICKING RESULTS FOR 31_ENSG00000067900_18:18690702 --> 31_ROCK1_2

OLIGO start len tm gc% any_th 3'_th hairpin seq

LEFT PRIMER 139 25 62.13 44.00 0.00 0.00 0.00 TCTCTTTTGCTTGTCTGGGGTTTTG

RIGHT PRIMER 303 25 61.94 48.00 0.00 0.00 0.00 GCAAATAGGACCACACCATGAGATG

PRODUCT SIZE: 165

PRIMER PICKING RESULTS FOR 31_ENSG00000121989_2:148676023 --> 31_ACVR2A

OLIGO start len tm gc% any_th 3'_th hairpin seq

LEFT PRIMER 89 25 62.68 48.00 0.00 0.00 0.00 CCTGAAAGGGAAACTCACAACCTCT

RIGHT PRIMER 282 25 62.00 44.00 14.67 10.52 0.00 ATATGCCAATCCTCTAGCCATGGTT

PRODUCT SIZE: 194

PRIMER PICKING RESULTS FOR 31_ENSG00000127914_7:91690637 --> 31_AKAP9

OLIGO start len tm gc% any_th 3'_th hairpin seq

LEFT PRIMER 160 25 61.81 44.00 0.00 0.00 0.00 GACAGAGTTGATGCGTGAGTCATTT

RIGHT PRIMER 334 25 61.52 44.00 0.00 0.00 0.00 TCTCTACCCTTAGTTGTCAATGGCA

PRODUCT SIZE: 175

PRIMER PICKING RESULTS FOR 31_ENSG00000169679_2:111428132 --> 31_BUB1

OLIGO start len tm gc% any_th 3'_th hairpin seq

LEFT PRIMER 54 25 61.30 44.00 0.00 0.00 0.00 GCAACTGTACATTCTCTCTTGACGT

RIGHT PRIMER 242 25 62.02 44.00 0.00 0.00 0.00 CCTGATTTTACCTTGAGCTGGCAAA

PRODUCT SIZE: 189

PRIMER PICKING RESULTS FOR 31_ENSG00000145675_5:67592099 --> 31_PIK3R1

OLIGO start len tm gc% any_th 3'_th hairpin seq

LEFT PRIMER 113 25 62.18 48.00 0.00 0.00 0.00 GTGGAAGATGATGAAGATTTGCCCC

RIGHT PRIMER 274 25 62.24 48.00 0.00 0.00 0.00 ATACACTACAGAGCAGGCATAGCAG

PRODUCT SIZE: 162

PRIMER PICKING RESULTS FOR 31_ENSG00000107643_10:49635181 --> 31_MAPK8

OLIGO start len tm gc% any_th 3'_th hairpin seq

LEFT PRIMER 52 25 58.35 36.00 0.00 0.00 0.00 AGTCATCTGTGTGGCTAATATTTCA

RIGHT PRIMER 247 25 61.70 44.00 0.00 0.00 0.00 AGCTCCTTCACAGTTTTCCCTCTAA

PRODUCT SIZE: 196

PRIMER PICKING RESULTS FOR 31_ENSG00000120251_4:158233974 --> 31_GRIA2

OLIGO start len tm gc% any_th 3'_th hairpin seq

LEFT PRIMER 94 25 61.90 40.00 6.90 0.00 0.00 TGAAAAGAAATGGCAAGTGACTGCT

RIGHT PRIMER 259 25 62.22 44.00 0.00 0.00 0.00 GCAAACCTGGTCTACAATGTCGTTT

PRODUCT SIZE: 166

PRIMER PICKING RESULTS FOR 31_ENSG00000169032_15:66774173 --> 31_MAP2K1

OLIGO start len tm gc% any_th 3'_th hairpin seq

LEFT PRIMER 78 25 61.98 48.00 0.00 0.00 0.00 CCTGTGTCAGTTCCCTCCTTTTCTA

RIGHT PRIMER 250 25 62.13 48.00 20.57 11.77 0.00 TCATACCGACATGTAGGACCTTGTG

PRODUCT SIZE: 173

PRIMER PICKING RESULTS FOR 31_ENSG00000070729_16:57950065 --> 31_CNGB1

OLIGO start len tm gc% any_th 3'_th hairpin seq

LEFT PRIMER 165 25 62.21 44.00 0.00 0.00 0.00 TCATTCCCTTTCTTTCAGACGGACA

RIGHT PRIMER 333 24 62.43 50.00 0.00 0.00 0.00 CAGAAACCACATCTCATGAACGCC

PRODUCT SIZE: 169

PRIMER PICKING RESULTS FOR 31_ENSG00000126934_19:4117562 --> 31_MAP2K2

OLIGO start len tm gc% any_th 3'_th hairpin seq

LEFT PRIMER 90 22 58.51 54.55 7.78 0.00 0.00 GGAGTCTCCCTAGGTAGCTAAC

RIGHT PRIMER 277 25 62.34 48.00 5.24 0.76 0.00 CAGCTCTGAGATCCTTTCGAAGTCA

PRODUCT SIZE: 188

PRIMER PICKING RESULTS FOR 31_ENSG00000138696_4:96073924 --> 31_BMPR1B

OLIGO start len tm gc% any_th 3'_th hairpin seq

LEFT PRIMER 91 25 61.92 48.00 0.00 0.00 0.00 TTCCTTATCATGACCTAGTGCCCAG

RIGHT PRIMER 242 25 61.26 44.00 0.00 0.00 0.00 TTTAGAAGTCTGTCACAGCCACATG

PRODUCT SIZE: 152

PRIMER PICKING RESULTS FOR 201_ENSG00000029363_6:136593183 --> 201_BCLAF1

OLIGO start len tm gc% any_th 3'_th hairpin seq

LEFT PRIMER 164 25 60.22 40.00 0.44 0.00 0.00 AAGGAGAATTGACATCTCACCAAGT

RIGHT PRIMER 332 26 58.60 34.62 0.00 0.00 0.00 TGCCTTGAGAAACTATATTTGGTACA

PRODUCT SIZE: 169

PRIMER PICKING RESULTS FOR 201_ENSG00000121741_13:20641499 --> 201_ZMYM2

OLIGO start len tm gc% any_th 3'_th hairpin seq

LEFT PRIMER 103 25 62.39 52.00 13.48 13.48 0.00 GATCTACTCTCTCACACCACAGCTG

RIGHT PRIMER 252 25 61.15 44.00 0.00 0.00 0.00 AGCAGCCATCAAATTACTAACCTCC

PRODUCT SIZE: 150

PRIMER PICKING RESULTS FOR 201_ENSG00000049323_2:33335707 --> 201_LTBP1

OLIGO start len tm gc% any_th 3'_th hairpin seq

LEFT PRIMER 130 25 61.97 48.00 0.00 0.00 0.00 GTTTCTTCCCAGAGTGACTCCTCTT

RIGHT PRIMER 323 25 61.91 48.00 0.00 0.00 0.00 GACTATACTCACGCTGAAAAGGTGC

PRODUCT SIZE: 194

PRIMER PICKING RESULTS FOR 2_ENSG00000100393_22:41513603 --> 2_EP300

OLIGO start len tm gc% any_th 3'_th hairpin seq

LEFT PRIMER 153 25 61.09 44.00 0.00 0.00 0.00 ATGAACAGTCCAGTAAATCAGCCTG

RIGHT PRIMER 321 25 61.93 48.00 0.00 0.00 0.00 GGTTTGGGTACTGCATATTCTGTCG

PRODUCT SIZE: 169

PRIMER PICKING RESULTS FOR 2_ENSG00000121989_2:148674942 --> 2_ACVR2A

OLIGO start len tm gc% any_th 3'_th hairpin seq

LEFT PRIMER 148 25 62.29 44.00 0.00 0.00 0.00 TTTGCCTGGAATGAAGCATGAGAAC

RIGHT PRIMER 333 25 61.64 44.00 9.85 0.00 0.00 AACTCTGAGGGATTTATAGCCAGCA

PRODUCT SIZE: 186

PRIMER PICKING RESULTS FOR 2_ENSG00000151617_4:148453812 --> 2_EDNRA

OLIGO start len tm gc% any_th 3'_th hairpin seq

LEFT PRIMER 103 25 61.85 44.00 0.00 0.00 0.00 TGCCATTGAAATTGTCTCCATCTGG

RIGHT PRIMER 283 25 62.30 48.00 0.00 0.00 0.00 CCTCCCTTCCCCAGTTAATTCCTTT

PRODUCT SIZE: 181

PRIMER PICKING RESULTS FOR 2_ENSG00000149269_11:77047300 --> 2_PAK1

OLIGO start len tm gc% any_th 3'_th hairpin seq

LEFT PRIMER 99 25 59.87 40.00 0.00 0.00 0.00 AGGTACACAGTCAACACTTACTGAA

RIGHT PRIMER 281 25 63.80 52.00 4.73 0.00 0.00 CATAGGCCTTTCGTGTCACAACCTC

PRODUCT SIZE: 183

PRIMER PICKING RESULTS FOR 2_ENSG00000117020_1:243776975 --> 2_AKT3

OLIGO start len tm gc% any_th 3'_th hairpin seq

LEFT PRIMER 161 25 61.84 40.00 0.00 0.00 0.00 AGACCGTTTGTGTTTTGTGATGGAA

RIGHT PRIMER 311 25 59.81 40.00 0.00 0.00 0.00 TCCTATCAAGAAATGGAGAACTGCT

PRODUCT SIZE: 151

PRIMER PICKING RESULTS FOR 2_ENSG00000180914_3:8809260 --> 2_OXTR

OLIGO start len tm gc% any_th 3'_th hairpin seq

LEFT PRIMER 98 23 63.59 56.52 9.96 0.00 0.00 CAGGTGCACATCTTCTCTCTGCG

RIGHT PRIMER 269 25 61.68 44.00 1.00 0.00 0.00 AGATCTTGAAGCTGATAAGGCCGTA

PRODUCT SIZE: 172

PRIMER PICKING RESULTS FOR 2_ENSG00000136997_8:128752998 --> 2_MYC

OLIGO start len tm gc% any_th 3'_th hairpin seq

LEFT PRIMER 142 25 62.97 48.00 0.00 0.00 0.00 GAATGTCAAGAGGCGAACACACAAC

RIGHT PRIMER 313 25 64.15 52.00 0.00 0.00 0.00 CTCTGCTTGGACGGACAGGATGTAT

PRODUCT SIZE: 172

PRIMER PICKING RESULTS FOR 28_ENSG00000135409_12:53823264 --> 28_AMHR2

OLIGO start len tm gc% any_th 3'_th hairpin seq

LEFT PRIMER 166 24 62.71 50.00 0.00 0.00 0.00 CCCACAGGCCAATATAAACCAGGT

RIGHT PRIMER 360 25 60.40 44.00 0.00 0.00 0.00 ATCCAGAGAACTCACTTCCATGATG

PRODUCT SIZE: 195

PRIMER PICKING RESULTS FOR 28_ENSG00000157764_7:140481478 --> 28_BRAF

OLIGO start len tm gc% any_th 3'_th hairpin seq

LEFT PRIMER 144 25 60.05 36.00 0.00 0.00 0.00 TTCTGTTTGGCTTGACTTGACTTTT

RIGHT PRIMER 307 25 61.99 44.00 0.00 0.00 0.00 TTACCATGCCACTTTCCCTTGTAGA

PRODUCT SIZE: 164

PRIMER PICKING RESULTS FOR 2_ENSG00000152270_11:14666156 --> 2_PDE3B_B

OLIGO start len tm gc% any_th 3'_th hairpin seq

LEFT PRIMER 158 22 62.97 54.55 0.00 0.00 0.00 GACTTCTTGGTGTGGCAGTGGT

RIGHT PRIMER 362 20 61.82 60.00 0.00 0.00 0.00 AGACCCACCAGACGAAGCTG

PRODUCT SIZE: 205

PRIMER PICKING RESULTS FOR 28_ENSG00000175634_11:67200425 --> 28_RPS6KB2_B

OLIGO start len tm gc% any_th 3'_th hairpin seq

LEFT PRIMER 34 23 62.67 56.52 0.00 0.00 0.00 CATCATCTACCGGGACCTCAAGC

RIGHT PRIMER 229 24 63.29 50.00 0.00 0.00 0.00 GCAGAGTCCAAAGTCGGTCAGTTT

PRODUCT SIZE: 196
